# Next-Generation Sequencing: Application in Liver Cancer—Past, Present and Future?

**DOI:** 10.3390/biology1020383

**Published:** 2012-08-31

**Authors:** Jens U. Marquardt, Jesper B. Andersen

**Affiliations:** 1 Department of Medicine I, Johannes Gutenberg University, Langenbeckstrasse 1, Mainz 55131, Germany; 2 Laboratory of Experimental Carcinogenesis, NCI/CCR, NIH, Bethesda, Maryland 20892, USA

**Keywords:** Hepatocellular carcinoma (HCC), Next-generation sequencing (NGS), personalized medicine, integrative genomics

## Abstract

Hepatocellular Carcinoma (HCC) is the third most deadly malignancy worldwide characterized by phenotypic and molecular heterogeneity. In the past two decades, advances in genomic analyses have formed a comprehensive understanding of different underlying pathobiological layers resulting in hepatocarcinogenesis. More recently, improvements of sophisticated next-generation sequencing (NGS) technologies have enabled complete and cost-efficient analyses of cancer genomes at a single nucleotide resolution and advanced into valuable tools in translational medicine. Although the use of NGS in human liver cancer is still in its infancy, great promise rests in the systematic integration of different molecular analyses obtained by these methodologies, *i.e.*, genomics, transcriptomics and epigenomics. This strategy is likely to be helpful in identifying relevant and recurrent pathophysiological hallmarks thereby elucidating our limited understanding of liver cancer. Beside tumor heterogeneity, progress in translational oncology is challenged by the amount of biological information and considerable “noise” in the data obtained from different NGS platforms. Nevertheless, the following review aims to provide an overview of the current status of next-generation approaches in liver cancer, and outline the prospects of these technologies in diagnosis, patient classification, and prediction of outcome. Further, the potential of NGS to identify novel applications for concept clinical trials and to accelerate the development of new cancer therapies will be summarized.

Key Points Describing the Potential and Challenges of NGS in Translational Liver Cancer Research

- Whole genome NGS promises to bring unprecedented sensitivity, single nucleotide resolution and multidimensional parallel detection of low abundant genetic variations coupled with epigenetic and transcriptional changes for the individual patient.
- Primary liver cancer is a disease with poor outcome which is increasing in frequency worldwide and shows limited drug-response in clinical trials. Past genomic studies have established that HCC is highly heterogeneous which may complicate the interpretation of NGS data.
- The breadth of data from NGS and the rapidity by which it is obtained promises through translational medicine to provide novel therapeutic options to the individual by deepening the insight into the mechanisms of the disease. However, the challenge in the analysis of NGS highlights not just the tumor heterogeneity but also the complexity, robustness and technical noise (*i.e.*, the difficulty in technical *versus* biological discrepancies).

## 1. Introduction

Cancers are caused by the accumulation of genomic and epigenomic alterations. Meaningful integration of these diverse biological layers is a main challenge for the understanding of complex diseases [1]. Exponential application of high-throughput methods over the past 10 years has significantly contributed to our understanding of cancer biology, diagnosis and therapy [2]. These novel technologies for the study of genomics, epigenomics, transcriptomics, and proteomics provide meaningful insights on the molecular features of different cancer subtypes [3]. However, this one-dimensional view on molecular alterations will most likely be advanced by a multidimensional integrative approach (*i.e.*, an integration of different molecular layers such as genomic, epigenomic or transcriptomic information in one analysis) using next-generation sequencing (NGS) analyses. Therefore, NGS technologies promise a global view on oncogenomics by facilitating integrative and efficient detection of genetic and epigenetic alterations in cancer at a single-base resolution. 

In the last few years the omics-age has experienced a revolution in NGS [4] and bioinformatic approaches [5] to analyze the challenging exponential growth in data. Technologies, software development and decreasing cost are literally changing by the minute to the point where we can obtain the complete sequence of a human genome roughly within a day at a price that is realistic for implementation to the clinic. Today, the challenge of translating and routinely applying NGS in the clinic is straightforward analysis and interpretation of the data to improve health care for the individual. Given the molecular heterogeneity observed in cancer, determination of causality between discovered variants and carcinogenesis will not be straight forward and requires a comprehensive *a priori* knowledge and understanding of the functional genome—an impediment that will take significant time and effort.

## 2. NGS, the Next Leap in Clinical Diagnostics

Translational oncology and integrative genomics are systems biology-based strategies suitable for decoding the human genome to determine the biological function and interaction of genes. The advantage of a multidimensional approach to translational oncology is the improved capacity to predict drug-response more accurately and to provide insight to complex clinical problems. The central hypothesis is that the application of a multidimensional approach will be the key to understanding many treatment-refractory diseases, such as primary liver cancers (e.g., HCC), and to future clinical success. It is imperative to future success that these methodologies are adapted to clinical trial designs and practice, particularly in drug-resistant cancers (e.g., primary liver cancers). Notably, genetic testing to guide cancer treatment, e.g., by assessing mutations in EFGR, KRAS, FLT3 and NPM is already established in other cancers. However, while the detection of these driver mutations are highly useful for clinical decision making, oncogene addiction such as observed in lung cancer and AML is less frequently observed in HCC. Therefore, detailed genetic information by NGS such as whole genome sequencing (WGS) or exome sequencing (WES) will provide an unprecedented multi-layered insight into the underlying biology of the disease. This offers the opportunity to detect less abundant genetic changes on a single nucleotide resolution which is essential to advance early diagnosis, identify prognostic markers and develop precision therapies for this disease.

To improve the genomic understanding of a heterogeneous cancer like HCC, and to identify curative treatment options, detailed information such as WGS from the individual patient may be a prerequisite for clinical success. Indeed, application of high resolution single nucleotide DNA sequencing of a complete primary tumor/normal genome from the same individual was first characterized from a patient with acute myeloid leukemia (AML) [6]. 

Already in 2008, Jones and colleagues combined genetic analyses and SAGE with massively parallel RNA sequencing by synthesis in pancreatic cancers and demonstrated that core pathways and regulatory processes can only be found by in-depth analyses [7]. Using this approach they found that on average pancreatic cancers contain 63 genetic alterations, most of them involved in a core set of 12 cellular signaling networks. Subsequently, the same group sequenced the genomes of seven primary pancreatic cancers to evaluate the sequence of malignant progression from primary cancer to metastatic disease [8]. The authors’ identified different clonal populations with the potential of generating distant metastases. Interestingly, it was clearly demonstrated that metastatic clones were evolved genetically from the original tumor. Consistently the majority of acquired somatic mutations that progress the generation of metastasis were already detectable within the primary tumors. When a quantitative analysis of the timing of the genetic evolution of pancreatic cancer was performed, duration of more than 15 years seemed to be required for the metastatic potential. Validation of these findings stems from a similar study where DNA sequencing was applied to 13 pancreatic cancers [9]. The authors’ confirmed that alterations of cancer genes were predominantly manifested in early stages of cancer development and not in advanced stages of disease. Key pathways affected by these genetic alterations involve telomere dysfunction and abnormal cell-cycle control (e.g., G1-to-S-phase transition with intact G2-M checkpoint). Further, the genetic alterations after cancer dissemination persisted, resulting in ongoing, parallel and even convergent evolution among different metastases. These NGS studies not only have important conceptual but also therapeutic implications, indicating new therapeutic windows for the potential curative treatment of pancreatic cancers.

Other studies focusing on the implementation of NGS in clinical diagnosis and consequently supportive of treatment decision-making proved extraordinarily effective in identifying therapeutic options, e.g., in the case of a patient with metastatic lung cancer, who initially was presented with a rare adenocarcinoma of the tongue [10]. Characterization of this cancer by genome and RNA sequencing prior to treatment suggested that the tumor progression was driven by aberrant expression of the *RET* oncogene and thus, the patient was placed on a targeted regimen with the potent multi-tyrosine kinase inhibitor sunitinib. Initial stable disease was followed by progressive lung cancer and subsequent tumor regression was then achieved by administration of sorafenib and sulindac, two drugs also identified in the initial analysis as potentially effective in this case. After disease stabilization, a recurrent metastasis progressed, new lesions developed and resistance to therapy was determined. Additional genome sequencing was then performed, demonstrating that the accrual of new somatic mutations was consistent with the observed drug resistance. This is a very important study showing the promise and influence of NGS-based technologies in understanding the underlying biology of primary cancers, development of therapeutic resistance and effective direct treatment decisions. 

## 3. Application of NGS in Liver Cancer

### 3.1. Genome Wide Associations Studies in Liver Cancer

The application of high-throughput technologies in HCC has a long standing tradition. Recently, the advent of these technologies for unraveling the genomics of liver diseases was initiated by the identification of different susceptibility loci using genome wide associations studies (GWAS) [11]. Identification of the interleukin 28B (*IL28B*) gene locus in the pathogenesis of hepatitis C virus (HCV) revolutionized the application of genomic data in prediction of therapeutic response [12,13,14]. The notion that genetic variation in the IL28B gene predicted treatment response and partially explained the observed ethnical disparity observed during standard HCV therapy opened the avenue of next-generation pharmacogenomics for diverse clinical applications. 

Subsequently, GWAS was successfully applied to indentify genetic variation associated with HCC development from different etiologies of viral background. A HCV-induced HCC investigation of 432,703 autosomal SNPs in 721 individuals of Japanese origin revealed eight SNPs with potential association to hepatocarcinogenesis [15]. Genetic variation in the MHC class I polypeptide-related sequence A gene *MICA* on 6p21.33 (rs2596542) was demonstrated to be associated with the progression from HCV to HCC. Although statistical significance of this association was overall low, protein levels of MICA could be detected in sera of affected patients with HCV-induced HCC [16]. Additionally, other SNPs such as rs1012068, an intronic SNP in the *DEPDC5* locus on chromosome 22, were associated with progression to HCC in a Japanese patient cohort [17].

The intronic SNP (rs17401966) located in *KIF1B* on chromosome 1p36.22 was demonstrated to be highly associated with hepatitis B virus (HBV)-induced HCC in 355 chronic HBV infected patients with HCC from China [18]. In addition to *KIF1B*, *UBE4B* and *PGD* showed potential association to hepatocarcinogenesis. Interestingly, the identified chromosomal region on 1p36.22 has been frequently implicated in cancer development in several malignancies such as colorectal cancer, breast cancer, neuroblastoma, and also in HCC [19]. Future investigation will demonstrate if these causal associations may have an impact for translational medicine in HCC patients [19].

### 3.2. Gene Expression Signatures in Liver Cancer

Experience with transcriptomics, *i.e.*, the generation of relevant gene expression signatures for diagnostic or therapeutic classification of HCC patients commenced almost a decade ago, and has been reviewed extensively [20,21,22,23]. In particular, the application of functional and comparative genomics as well as the systematic application of integrative high-throughput approaches have greatly advanced our understanding of hepatocarcinogenesis and led the identification of several relevant genes within the landscape of molecular alterations in HCC [24,25,26,27]. Despite these great efforts, clinical translation of these findings for everyday medical practice such as observed in other cancers remains to be demonstrated [28]. One potential reason is that sensitivity of imaging techniques (e.g., magnetic resonance imaging and computer tomography) significantly improved diagnosis of HCC and in recent guidelines biopsies are no longer mandatory to establish the diagnosis [29]. Therefore, systematic validation of generated signatures in independent patient cohorts is virtually impossible. Further, traditionally one-dimensional approaches (*i.e.*, genomics/transcriptomics/epigenomics) have been applied to HCC patients and multilevel integration of the different molecular layers remains the exception. We have recently performed an integrative transcriptomic and epigenomic profiling to generate a de-methylation response signature that could be useful to identify patients that are likely to benefit from therapeutic agents that target the cancer epigenome in HCC [30]. Also, a group from Heidelberg integrated genome-wide methylation profiling with array-based CGH, and gene expression data from patients with HCC [31]. As a result, the author’s identified three new potential tumor suppressor genes (*PER3*, *IGFALS*, *PROZ*) downregulated in human HCCs compared to peritumoral and normal liver tissues. These studies illustrate the potential of integrative multi-layer approaches to facilitate the identification of promising new targets in liver cancer and clear the way for NGS approaches. 

### 3.3. Whole Genome/Exome Sequencing in Liver Cancer

As previously mentioned DNA sequencing of hematological cancers such as AML has proven a success [32,33]. However, implementation of NGS to solid tumors like HCC provides additional challenges as the proportion of normal cells or the stromal composition within a given sample contributes to the genomic signature and therefore may require additional coverage (*i.e.*, read depth). Also, HCC often arise in the background of a chronically diseased liver with underlying cirrhosis, fibrosis, HBV or HCV infection which may complicate the tumor/normal variant discovery when compared to the peritumoral liver tissue or even blood. Prospectively, a skin biopsy taken from the patient at diagnosis may be an alternative option.

The number of studies where NGS technologies have been applied to investigate HCC is so far limited. The course of translating the last decade of genomic profiling into the sequencing era is however increasingly entering practice and will likely become standard as cost decrease and bioinformatic tools become easier to use. The first primary liver cancer genome was sequenced in 2011 from a Japanese male diagnosed with HCV positive HCC [34]. Here, massively parallel 50 base pair paired end reads and WES were used to analyze the genome from the tumor and lymphocytes obtained from the same patient, revealing a total of 11,731 tumor-enriched somatic mutations. Interestingly, the prevalence of somatic alterations was largely in intergenic regions and only 88 substitutions or small insertions and deletions were validated, including *TP53* and *AXIN1*. Clinically, NGS has proven powerful in the detection of viral infection (e.g., HCV) in liver biopsies [35]. Deep sequencing has also been applied in a longitudinal analysis of the viral evolution following early viremia in four asymptomatic acute HCV infected patients where blood samples were collected over the initial 24 weeks [36]. To develop vaccines against e.g., HCV, understanding of selective pressure on the viral population/genomes following infection is needed. Therefore, information on the founder strain that effectively infected the host/patient and the primary infection which either results in clearance or drives disease progression, causing chronic infection and lastly liver disease, are important parameters to effectively control. Recently, a couple of studies applied WES to cases with primarily HCV-related [37] or alcohol-associated [38] HCC. In the first study, the authors sequenced 10 HCV-related HCC cases and compared to normal tissues from the same patients [37]. Although, an average of ~43 mutations were identified per tumor only five genes (*CTNNB1*, *TP53*, *ARID2*, *DMXL1* and *NLRP1*) were determined to have recurrent mutations in more than two tumors. Re-sequencing of these genes was performed in a larger cohort of HCC cases which were subdivided into known risk factors (HBV, HCV, mixed or non-viral background). Interestingly, the novel HCV-related mutation in *ARID2* (6/43, 14%) was correlated with mutations in *CTNNB1* (13/43, 30%) but mutually exclusive with *TP53* mutations known to be associated with HBV infection. Similarly, in a cohort of 24 HCCs analyzed by WES, somatic mutations of a related chromatin remodeling gene *ARID1A* (16.8%) was predominantly associated with patients with a high intake of alcohol [38].

Another study used paired-end sequencing of 25 individuals with HCC from viral (*i.e.*, HBV and HCV) and non-viral etiology, including two sets of multicentric tumors in comparison to matched normal lymphocytes [39]. Overall average genome coverage between 30–40× could be obtained. The number of somatic substitutions, indels and rearrangements did not vary between different viral-related (HBV- and HCV) HCCs. However, alcohol drinking and multiple liver nodules were associated with specific somatic substitution patterns. Interestingly, no overlap in somatic mutations could be detected in multicentric tumors underlining the dramatic genetic heterogeneity observed in other cancers (e.g., kidney and breast) and indicates that the clonal origin of these tumor pairs is distinct [40,41]. Statistical and functional analyses showed that across all 27 HCC genomes, more than 2,000 (75.9 per tumor) protein-altering point mutations, including missense mutations, nonsense mutations, short coding indels and splice-site mutations. Consistently, *TP53*, *CTNNB1* and *EGFR* genes were frequently mutated in HCC. Additionally, multiple chromatin regulators, including *ARID1A*, *ARID1B*, *ARID2*, *MLL and MLL3*, were mutated in around half of all tumors, confirming the crucial role of the ARID family in hepatocarcinogenesis. Moreover, Hepatitis B virus genome integration was investigated. HBV integration in the TERT locus in a high clonal proportion was observed indicating that this event may confer growth advantage in the early phase of HBV-related liver carcinogenesis. Another recent study focused on the importance of HBV viral integration for hepatocarcinogenesis and validated the importance of TERT [42]. Massively parallel sequencing of 81 HBV-positive and 7 HBV-negative hepatocellular carcinomas (HCCs) and adjacent normal tissues was performed. The authors’ found that HBV integration observed at a higher incidence in tumors compared to normal tissue and associated with induction of chromosomal instability and Copy-number variations (CNVs). Furthermore, recurrent HBV integration events could be demonstrated and subsequently been validated by RNA-Seq and Sanger sequencing. In summary, well known genes associated to cancer development such as *TERT*, *MLL4* and *CCNE1* were demonstrated to be upregulated in tumor tissue. Finally, the author’s could demonstrate that the number of HBV integrations is associated with patient survival.

### 3.4. RNA Sequencing in Liver Cancer

In relation to NGS, at present we have predominantly discussed WGS, however, RNA sequencing is the most direct comparative NGS approach to conventional microarray technologies to comprehensively characterize the transcriptome while concomitantly obtaining information about genetic alterations (e.g., SNVs, gene fusion, *etc.*). However, thus far, RNA sequencing was only used in one study to investigate the HCC transcriptome in 10 matched HBV-related HCC cases, identifying a total of 1,378 differentially expressed genes [43]. Interestingly, downstream enrichment analysis of these genes showed a significant correlation with chromosome location on 8q21.3–24.3. Indeed, Woo *et al*. showed in 139 HCC patients in a combined analysis of copy number alterations and gene expression that genes located on chromosome 8q were the most predictive of overall survival and that 22/50 potential driver genes were located in this region [44].

### 3.5. NGS in Cholangiocellular Carcinoma

The application of NGS technology for other primary liver cancers is even more limited. Only one study applied WES for the study of Opisthorchis viverrini-related cholangiocarcinoma (CCA) [45]. *O. viverrini* is a trematoda endemic in Thailand, Laos und Malaysia associated with the development of CCA that constitutes a major public health concern in these areas. The authors’ performed whole-exome sequencing of eight *O. viverrini*-related tumors and matched normal tissue and validated 206 somatic mutations in 187 genes using Sanger sequencing. Frequent somatic mutations could be revealed in key genes such as *TP53*, *KRAS* and *SMAD4*. Additionally, alteration in 10 previously unrecognized genes, included inactivating mutations in *MLL3*, *ROBO2*, *RNF43* and *PEG3*, and activating mutations in the *GNAS* oncogene could be detected. This study does not only improve our understanding of the landscape of mutations in CCA, it underlines the importance of genes involved in histone modification for liver cancers other than HCC.

### 3.6. Other Applications of NGS in Liver Cancer

The concept that so called cancer stem cells (CSCs) or tumor-initiating cells (TICs) are exclusively responsible for the development and progression of many tumors including HCC is growing [46]. Already several years ago a group from Hong Kong demonstrated that the mesenchymal stem cell marker CD90 might be a potential marker of liver CSCs [47,48]. As a new application for NGS technology the authors’, in continuation of their previous work, employed RNA-Seq for the detailed characterization of the putative CD90 liver CSCs in comparison to non-tumorous liver tissues [49]. FACS-sorted CD90 cells from three different HCCs as well as adjacent non-tumorous human liver tissues were subjected to pair-end sequencing analysis. A total of 500 genes were identified to be differentially expressed between the putative CSCs and non-tumorous human liver tissues. Consistently, these genes were involved in pro-carcinogenic pathways such as inflammation, drug resistance and lipid metabolism. Among the identified genes, the commonly used HCC marker glypican-3 (*GPC3*) could be detected. Overall, this study serves as a proof-of-principle for the feasibility of performing RNA-Seq in a wide range of different applications in liver cancer research.

## 4. Conclusions

According to the commonly accepted dogma in cancer research, hallmarks of cancer include at least eight conserved biological capabilities [50]. These general properties of cancer cells are acquired during the multistep malignant transformation and include unlimited proliferation potential, evading growth suppressors and immune response, increased energy metabolism, resistance to cell death, replicative immortality, angiogenesis, as well as the potential to grow invasively and a metastatic potential [50]. In primary liver cancer and other malignancies the underlying genetic diversity is fostered by chronic inflammation of a permissive tumor microenvironment. 

Conceptual and technical progress in the last decade has greatly advanced our limited understanding of tumor biology. Increasing awareness of the genetic complexity and intratumoral heterogeneity remains a major challenge in the translational application of high-throughput data for individualized medicine. The future of next-generation approaches holds great promise for a better integration of multiple molecular layers to ultimately have a more meaningful impact for clinical applications. However, to achieve this challenging goal and to fully utilize the potential of NGS approaches for the understanding of the liver cancer genome, systematic application of genome-wide analyses into clinical trials will be necessary. In this context, high-throughput analyses should be adapted for diagnostic and prognostic classification, dissecting the mechanism of acquired resistance and predicting recurrence to ultimately contribute to treatment decisions and new drug development [3]. If this endeavor could be achieved, it is highly possible that NGS technology will continue to transform cancer research, leading to a comprehensive understanding of individual tumor genetics. However, this goal will depend on the generation of better computational analyses to identify changes of biological relevance within the continuously growing flood of genomic data. Furthermore, collection of the diverse information in large databases to connect genomic findings with clinical parameters will be of central importance. The near future will show if liver cancer is braced for the NGS era.
